# Celebrating the first anniversary of BMC Global and Public Health

**DOI:** 10.1186/s44263-024-00086-x

**Published:** 2024-07-31

**Authors:** Ben Cranfield, Gen Li, Gerrit John-Schuster

**Affiliations:** 1 Springer Nature, BioMed Central, London, UK; 2 Springer Nature, BioMed Central, Shanghai, China; 3 Springer Nature, BioMed Central, New York, NY USA

*BMC Global and Public Health published its first articles one year ago in July 2023. We are looking back at an exciting year of content, guest-edited collections and support from the research communities*.

We are delighted to be celebrating our journal’s first anniversary; an important milestone that we would not have been able to achieve without our authors, reviewers, editorial board members, guest editors, and journal team. The trust and support that we have received from the global and public health research communities has been, and continues to be, invaluable.

We have published more than 70 articles in our first year, including original research, and invited reviews, perspectives, and comments. Our authors represent a global research community covering all continents and collaborating across regions and countries to address shared and setting-specific health challenges. Our published content already covers a diverse range of research areas including infectious diseases, cancer care, reproductive, maternal and child health, social determinants of health, nutrition, mental health, health systems, public health policy, and health and care inequities. We envision that all studies will be impactful within their respective fields and settings as they aim to address disparities and to advance relevant Sustainable Development Goals. Many of the articles that we published have involved people with lived experiences, health care providers, policy makers, and other important stakeholders. We look forward to recognizing and publishing more voices from the communities that global and public health studies serve.

Under the guidance of two guest editors, our collection on identifying people with tuberculosis (TB) and linking to care (https://www.biomedcentral.com/collections/identifying-people-with-tb) has attracted more than ten articles covering timely and diverse topics that hold relevance even beyond TB. These studies explore active case finding approaches and their cost-effectiveness, the use of artificial intelligence (AI) in diagnosis, subclinical and pediatric TB, malnutrition as a major comorbidity of TB, and preference research among people and communities most affected by TB, among others. For a comprehensive overview, we recommend reading our recent editorial that summarized this inspiring content [1].

The perceptions and experiences of the stigma associated with TB, HIV, and COVID-19 were explored in another collection that we opened for research on stigma and mental health issues associated with infectious diseases (https://www.biomedcentral.com/collections/stigma-mh-infectious). In this collection, two studies report different TB stigma intervention development projects from South Africa and Indonesia [2, 3]. This work highlights how a participatory intervention model has been successfully applied to provide psychosocial support to people with TB. Although there are encouraging signs for interventions to reduce barriers in public health caused by stigma, there remains a significant physical challenge in expanding healthcare services to reach the needs of remote populations. This narrative is emphasized in recent publications in our collection aiming to explore the implementation of cancer strategies in primary care to support early diagnosis (https://www.biomedcentral.com/collections/ICSPC). These articles showcase research that highlights the challenges in colorectal cancer screening in Atlantic Canada and opportunities for improving HPV testing and vaccination in remote communities in Ecuador [4, 5]. Furthermore, Whitaker and colleagues present a timely perspective on the challenges in understanding inequities in help-seeking for possible cancer symptoms [6]. Reducing inequities in cancer outcomes remains a global health priority.

Our latest collections aim to cover emerging topic areas of interest in global public health, including geographic challenges to providing equitable healthcare (https://www.biomedcentral.com/collections/GCEH), loneliness and social isolation among aging populations (https://www.biomedcentral.com/collections/LPH), and health inequalities in sexual and gender minority groups (https://www.biomedcentral.com/collections/HI). With the help of guest editors, we have also curated an exciting pipeline of collections to be launched in the coming months — please keep an eye out for updates on our website.

Our editorial board members are integral to the journal’s success and growth as they support us in various day-to-day efforts such as manuscript submissions, collection topics, and other relevant journal decisions and activities, including community outreach and soliciting content. Since last year, our editorial board has significantly expanded its size and scope, bringing together a diverse and dynamic group of people from all over the world. We currently have the support of an editorial board consisting of 23 members (11 female and 12 male researchers), from 11 different countries across five continents, covering areas such as infectious and non-communicable disease epidemiology, maternal and child health, environmental and occupational health, genetic screening, disease modeling, health economics, social determinants of health, and more. We look forward to growing our editorial board as the journal continues to develop.

We attended some exciting conferences in the past year. At the *Nature Conference*’Advancing Health with AI’ in April 2024, we joined conversations with researchers and editors from other journals to discuss the potential of AI to facilitate health equity. We also learnt about the latest applications of AI in key global health fields such as antibiotic resistance testing and low-cost digital interventions in resource-limited settings. At the meeting of the *Society for Epidemiologic Research*, we learnt about the latest methods for data modeling in epidemiology studies and how this community explored causal associations of exposure and disease outcomes. Additionally, this year we are excited to be attending the *European Conference for Mental Health* and the *European Public Health Conference*, where we look forward to learning about novel and innovative research.

We have enjoyed our first year and the opportunities to develop a journal that will contribute to the dissemination of high-quality global public health research and opinions. We are incredibly grateful to our stakeholders and readers for their interest and support in the journal.

## Data Availability

Not applicable.
